# The nicotinic acetylcholine receptor gene family of the silkworm, *Bombyx mori*

**DOI:** 10.1186/1471-2164-8-324

**Published:** 2007-09-15

**Authors:** Ya-Ming Shao, Ke Dong, Chuan-Xi Zhang

**Affiliations:** 1 Institute of Insect Science, Zhejiang University, Hangzhou, Zhejiang, 310029, China; 2 Department of Entomology and Neuroscience Program, Michigan State University, East Lansing, MI 48824, USA

## Abstract

**Background:**

Nicotinic acetylcholine receptors (nAChRs) mediate fast synaptic cholinergic transmission in the insect central nervous system. The insect nAChR is the molecular target of a class of insecticides, neonicotinoids. Like mammalian nAChRs, insect nAChRs are considered to be made up of five subunits, coded by homologous genes belonging to the same family. The nAChR subunit genes of *Drosophila melanogaster*, *Apis mellifera *and *Anopheles gambiae *have been cloned previously based on their genome sequences. The silkworm *Bombyx mori *is a model insect of Lepidoptera, among which are many agricultural pests. Identification and characterization of *B. mori *nAChR genes could provide valuable basic information for this important family of receptor genes and for the study of the molecular mechanisms of neonicotinoid action and resistance.

**Results:**

We searched the genome sequence database of *B. mori *with the fruit fly and honeybee nAChRs by tBlastn and cloned all putative silkworm nAChR cDNAs by reverse transcriptase-polymerase chain reaction (RT-PCR) and rapid amplification of cDNA ends (RACE) methods. *B. mori *appears to have the largest known insect nAChR gene family to date, including nine α-type subunits and three β-type subunits. The silkworm possesses three genes having low identity with others, including one α and two β subunits, α9, β2 and β3. Like the fruit fly and honeybee counterparts, silkworm nAChR gene α6 has RNA-editing sites, and α4, α6 and α8 undergo alternative splicing. In particular, alternative exon 7 of Bmα8 may have arisen from a recent duplication event. Truncated transcripts were found for Bmα4 and Bmα5.

**Conclusion:**

*B. mori *possesses a largest known insect nAChR gene family characterized to date, including nine α-type subunits and three β-type subunits. RNA-editing, alternative splicing and truncated transcripts were found in several subunit genes, which might enhance the diversity of the gene family.

## Background

The nicotinic acetylcholine receptor (nAChR) is a ligand-gated ion channel (LGIC) that mediates fast synaptic cholinergic transmission in the insect central nervous system [1]. A nAChR is formed by five subunits arranged around a central pore that is permeable to cations [2]. These subunits are encoded by multiple α- and β subunit genes. Diverse nAChRs, differing in subunit composition, have different electrophysiological and pharmacological profiles [3]. Agonists, such as acetylcholine, bind to the N-terminal extracellular domain of the subunits at a site formed by six loops (Loop A-F) located at subunit interfaces. The N-terminal extracellular domain also contains a Cys-loop, characteristic of cys-Loop LGICs, which consists of two disulphide-bond forming cysteines separated by 13 residues [2]. Each subunit also has four transmembrane regions (TM1–TM4). The subunits with two vicinal Cys at loop C which are involved in acetylcholine binding are classified into α type, whereas other subunits lacking the two Cys residues are regarded as non-α type [4]. Phosphorylation sites are found in a long intracellular loop between TM3 and TM4, which may modulate the receptor activity [5,6].

The genomes of *Drosophila melanogaster*, *Anopheles gambiae*, *Apis mellifera *have been completely sequenced and their nAChR gene families have been annotated. *D. melanogaster *has seven α and three β subunits [7], *A. gambiae *has nine α and one β [8], and *A. mellifera *possesses nine α and two β [9]. Mammals and chicken have 16 and 17 nAChR subunits respectively [10]. Compared with vertebrates, insects seem to have a small nAChR gene family. However, recent studies showed that insects may enhance the functional diversity through alternative splicing of exons and RNA editing of transcripts [7,9]. In *Drosophila*, alternative splicing leads to the loss of some exons or retention of introns in mature nAChR mRNA [7].

Although study of insect nAChRs is still at an early stage, researchers have exploited nAChR as an insecticide target for a long time [11]. To defend against insects and other herbivores, plants synthesize compounds having insecticidal activities. Nicotine extracted from tobacco, for example, has been used as a commercial insecticide extensively, which is a potent agonist of nAChR [11]. Neonicotinoids are a group of insecticides, including imidacloprid, thiamethoxam [12]. These insecticides exhibit low effective dosage, selective toxicity for insects over vertebrates, wide insecticidal activity, and excellent uptake in plants [13]. Since the first neonicotinoid insecticide imidacloprid was put into market, it has gained the fastest growing sales of any insecticide worldwide [12].

The silkworm, *Bombyx mori*, producing silk, is an important beneficial insect in many countries. Recently this insect gained more attention because the silkworm bodies can express foreign proteins after being infected with recombinant baculovirus [14]. *B. mori *is also a good model organism because of its large body size, ease of rearing [15], and the completion of the whole genome sequence [16,17]. *B. mori *belongs to Lepidoptera, the second largest order of Insecta, possessing about 200 thousand species. Most insects of this order are herbivores and eat plants at the larval stage and many are agricultural pests except for the silkworm. Up to date, five α and one β subunits from *Heliothis virescens*, one α and one β from *Manduca sexta*, one α from *Chilo suppressalis *and one β subunit from *Spodoptera exigua *are deposited in GenBank. In addition, three alternative transcripts of *B. mori *α6 were reported [18]. Despite the agricultural importance of lepidopteran insects, the nAChR subunit gene family from this order remains unclear. In this report, we report the molecular characterization of the nAChR gene family from the silkworm.

## Results

### Twelve candidate nAChR subunit genes in the *B. mori *genome

**Figure 1:**
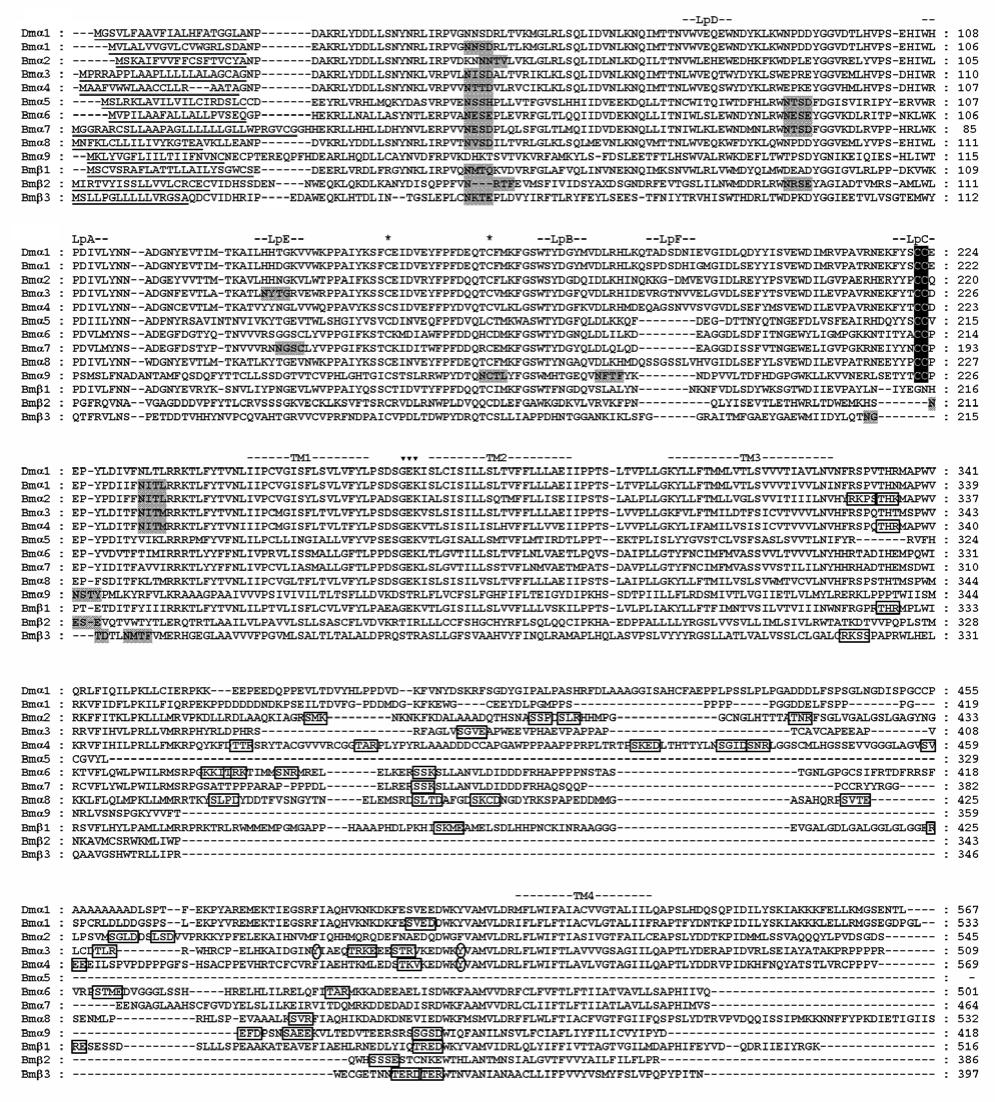
**Alignment of *B. mori *nAChR subunit protein sequences**. *D. melanogaster *α1 is included for comparison. N-terminal signal peptides are underlined. Position of loops (LpA-F) involved in ligand binding and transmembrane motifs (TM1–4) forming the ion channel are indicated. Sites of Cys residues involved in Cys-loop are marked with asterisks and the vicinal Cys residues characteristic of α-type are showed in white letters. GEK motif associated with cation selectivity is indicated by inverted triangles. Putative N-glycosylation sites are highlighted in shade. Potential PKA, PKC, and CK2 phosphorylation sites are boxed and potential tyrosine kinase phosphorylation sites are enclosed in ovals.

We searched the *B. mori *genome sequence database for nAChR subunit genes by tBlastn [19]. Twelve candidate genes were identified. Through RT-PCR and RACE analyses, expression of all 12 nAChR subunit genes was confirmed and complete open reading frames (ORFs) were obtained except Bmα7. A 121 bp sequence encoding the signal peptide at the N-terminus of Bmα7 was obtained from an EST sequence [GenBank: BB982760] by BLAST search [20] and the complete ORF of Bmα7 was then assembled. Alignment of predicted protein sequences (Figure 1) shows that all these subunits possess two Cys separated by 13 residues, typical of LGIC subunits, as well as six conserved Loops (Loops A-F) located at the N-terminal domain. Nine of them, having two vicinal Cys at Loop C, belong to type α, whereas the other three lack this motif and are therefore of type β. All subunits carry a predicted signal peptide at the N-terminus, and all but one (Bmα5) possess four transmembrane motifs (TM1–TM4) at the C-terminal domain, which are involved in forming an ion channel. Bmα5 has only three transmembrane regions (TM1–TM3). Loops between TM3 and TM4 were highly variable and have predicted phosphorylation sites. Putative N-glycosylation sites are present in the N-terminal extracellular domains of all nAChR subunits [21].

**Figure 2:**
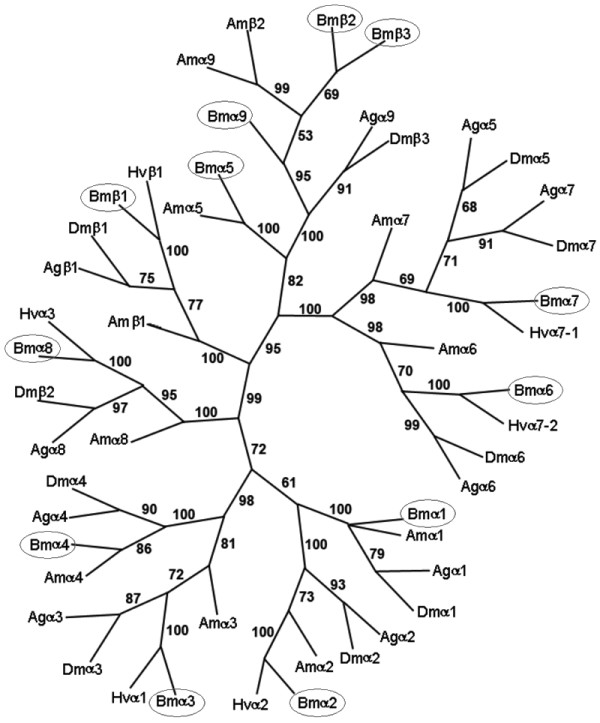
**Phylogenetic relationship of insect nAChR subunits**. The bootstrap 50% majority-rule consensus trees were made with the maximum parsimony method (PAUP, Version 4.0) using multiple alignments of amino acid sequences. Statistical support (percentage) for each node was evaluated by bootstrap analysis with 1,000 replicates. *B. mori *nAChRs cloned by us are enclosed by ovals. The nAChR subunits shown in the tree are as follows including GenBank accession numbers: *Anopheles gambiae *Agα1 [GenBank: AAU12503], Agα2 [GenBank: AAU12504], Agα3 [GenBank: AAU12505], Agα4 [GenBank: AAU12506], Agα5 [GenBank: AAU12508], Agα6 [GenBank: AAU12509], Agα7 [GenBank: AAU12511], Agα8 [GenBank: AAU12512], Agα9 [GenBank: AAU12513], Agβ1 [GenBank: AAU12514]. *Drosophila melanogaster *Dmα1 [GenBank: CAA30172], Dmα2 [GenBank: CAA36517], Dmα3 [GenBank: CAA75688], Dmα4 [GenBank: CAB77445], Dmα5 [GenBank: AAM13390], Dmα6 [GenBank: AAM13392], Dmα7 [GenBank: ABO26063], Dmβ1 [GenBank: CAA27641], Dmβ2 [GenBank: AAF56304], Dmβ3 [GenBank: CAC48166]. *Apis mellifera *Amα1 [GenBank: AAY87890], Amα2 [GenBank: AAS48080], Amα3 [GenBank: AAY87891], Amα4 [GenBank: AAY87893], Amα5 [GenBank: AAS75781], Amα6 [GenBank: AAY87895], Amα7 [GenBank: AAR92109], Amα8 [GenBank: AAM51823], Amα9 [GenBank: AAY87896], Amβ1 [GenBank: AAY87897], Amβ2 [GenBank: AAY87898]. *Heliothis virescens *Hvα1 [GenBank: AJ000399], Hvα2 [GenBank: AF096878], Hvα3 [GenBank: AF096879], Hvα7-1 [GenBank: AF143846], Hvα7-2 [GenBank: AF143847], Hvβ1 [GenBank: AF096880].

Most *B. mori *nAChRs show high levels of sequence identities with the counterparts of *D. melanogaster*, up to 86% (Table 1). However, Bmα5 shares a low sequence identity with *D. melanogaster *α5, although it shows the 48% amino acid identity with *A. mellifera *α5. We constructed a phylogenetic tree comprising 39 insect nAChR subunit protein sequences (Figure 2). In fruit fly, mosquito and honeybee, nAChR subunits α1, α2, α3, α4, α6, α8, and β1 have strong corresponding relationships. Silkworm also has counterparts of these subunits, which are consequently named according to the existing nomenclature. However, subunits Bmα5 and Bmα7 are difficult to identify. In *A. mellifera*, α7 was named because its N-terminal domain shared more identity with *D. melanogaster *α7 and another was named α5 [9]. We named *B. mori *α5 and α7 following the nomenclature of *A. mellifera *because they had strong corresponding relationships. *D. melanogaster *β3 and *A. gambiae *α9 had low amino acid identities with other gene family members. Recently, *A. mellifera *β2 and α9 were also found having low sequence identities with other members of the nAChR family, and the two genes are located closely in the genome at a distance of 10 kb. It was suggested that the two genes arose from a recent evolutionary duplication event [9]. As shown in Table 1, three *B. mori *subunits, Bmα9, Bmβ2, and Bmβ3, have low sequence similarities with other nAChR subunits. Phylogenetic analysis indicates that Bmβ2 and Bmβ3 are clustered into one branch, suggesting that Bmα9 had diverged before the two β subunits separated evolutionarily. Bmα9 shows 27% sequence identity with *A. mellifera *α9 and 21% with *A. gambiae *α9, respectively. Bmβ2 and Bmβ3 show equal sequence identities with *A. mellifera *β2, 17%.

**Table 1:** Percentage identity/similarity between putative *B. mori *and *D. melanogaster *nAChR subunits

Subunit	Bmα1	Bmα2	Bmα3	Bmα4	Bmα5	Bmα6	Bmα7	Bmα8	Bmβ1	Bmα9	Bmβ2	Bmβ3
Bmα1	--	53/68	54/69	48/63	25/39	33/49	33/50	54/69	37/53	15/32	13/28	11/26
Dmα1	**74/82**	51/66	53/66	49/63	23/36	31/48	32/47	52/66	38/52	15/30	11/26	11/24
Bmα2	53/68	--	48/65	43/60	22/36	31/50	32/50	49/66	37/54	15/31	12/26	10/25
Dmα2	50/64	**77/83**	48/62	42/57	22/35	30/48	32/48	47/65	36/53	14/31	12/26	10/25
Bmα3	54/69	48/65	--	62/71	26/40	34/50	36/51	55/70	40/56	18/34	11/29	13/28
Dmα3	37/47	34/45	**52/56**	43/51	17/26	22/35	23/34	37/47	28/38	11/22	7/19	8/18
Bmα4	48/63	43/60	62/71	--	22/35	31/47	30/46	49/63	36/51	15/29	10/26	11/23
Dmα4	49/62	44/60	62/71	**70/77**	22/36	30/47	31/48	50/64	37/53	15/29	11/26	11/23
Bmα5	25/39	22/36	26/40	22/35	--	27/41	28/44	24/38	24/40	16/34	14/29	11/25
Dmα5	23/34	23/34	23/33	21/32	**17/26**	42/49	48/53	21/34	21/33	9/20	8/18	7/17
Bmα6	33/49	31/50	34/50	31/47	27/41	--	65/75	32/52	32/52	15/33	14/29	12/26
Dmα6	32/49	32/49	34/50	31/46	28/44	**79/87**	66/76	33/50	32/51	16/34	14/30	12/29
Bmα7	33/50	32/50	36/51	30/46	28/44	65/75	--	32/52	32/50	15/33	13/30	12/27
Dmα7	32/47	32/50	34/48	29/45	25/39	60/71	**70/77**	31/47	29/48	13/29	11/26	10/23
Bmα8	54/69	49/66	55/70	49/63	24/38	32/52	32/52	--	37/55	16/32	12/28	12/26
Dmβ2	52/69	47/64	55/71	49/62	25/38	33/50	33/52	**72/85**	37/54	16/32	12/29	13/28
Bmβ1	37/53	37/54	40/56	36/51	23/39	33/52	33/50	37/54	--	15/33	11/28	12/26
Dmβ1	37/53	35/53	40/57	35/51	24/40	32/52	32/50	37/55	**86/89**	15/33	12/29	12/26
Bmα9	15/32	15/31	18/34	15/29	16/34	15/33	15/33	16/32	15/33	--	24/45	21/39
Dmβ3	17/36	16/35	17/36	16/33	17/30	17/35	17/36	17/35	16/33	19/38	15/32	13/27
Bmβ2	13/28	12/26	11/29	10/26	14/29	14/29	13/30	12/28	11/28	24/45	--	22/40
Bmβ3	11/26	10/25	13/28	11/23	11/25	12/26	12/27	12/26	12/26	21/39	22/40	--

### Gene structure

**Figure 3:**
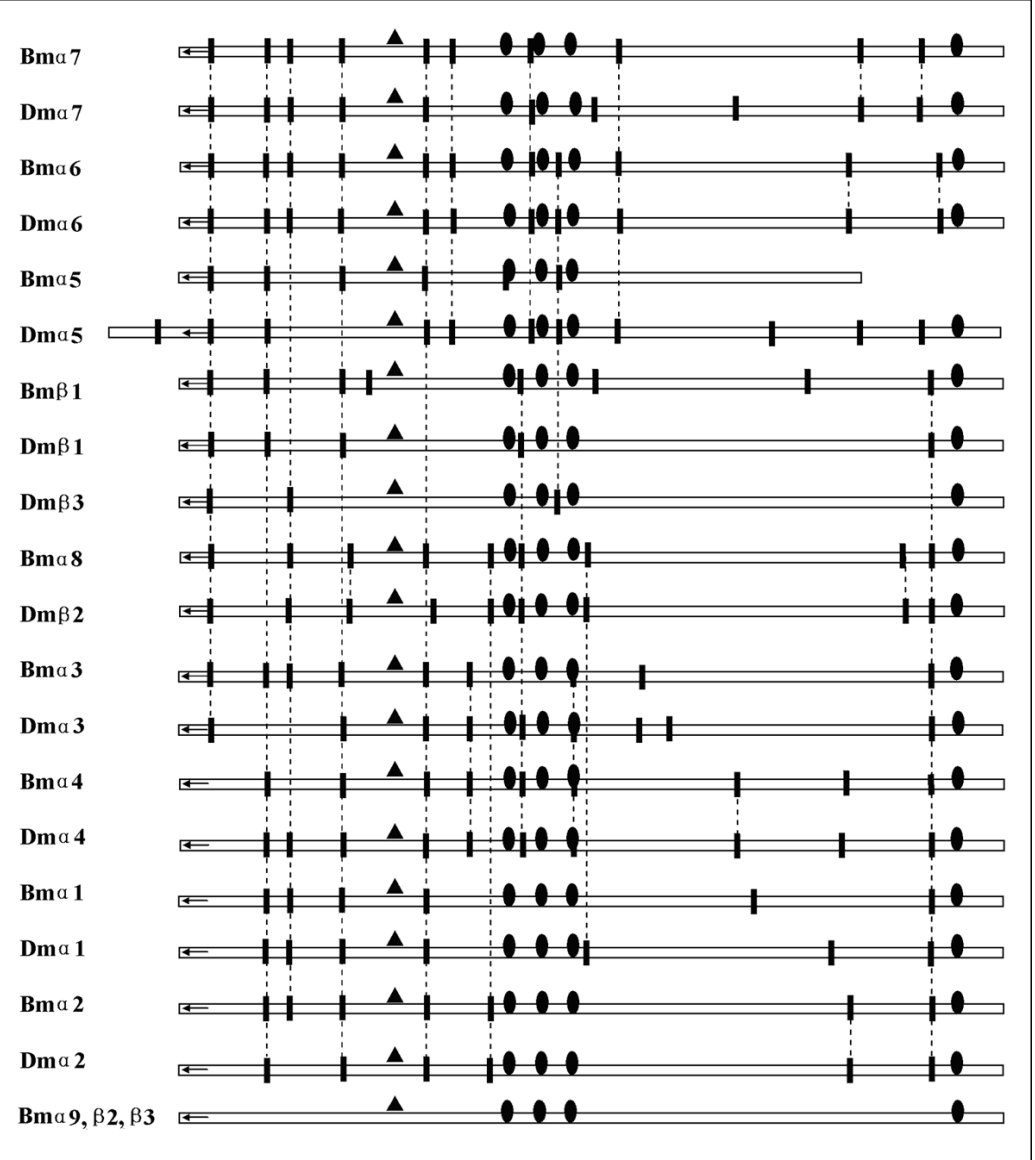
**Comparison of exon junctions of *Bombyx mori *and *Drosophila melonogaster *nAChRs**. N-terminal signal leader peptides are symbolized by arrows. Exon junctions are indicated by vertical bars. Sites of transmembrane motifs are indicated by ovals and Cys-loops are showed by triangles. Identical exon boundaries are showed by dotted lines.

Figure 3 shows that only subunits α6 of *Bombyx *and *Drosophila *share the same exon-intron boundaries. Another pair, subunit Bmα8 and its *Drosophila *counterpart, β2, only differs in one exon boundary. Bmα3 and Bmα7 possess the same number of exons with their *Drosophila *counterparts. Bmα2 and Bmβ1 of *Bombyx *possess more exons than their *Drosophila *counterparts and α1, α4, and α5 of *Bombyx *have fewer exons than their *Drosophila *counterparts. Surprisingly, three subunits of *B. mori*, Bmα9, Bmβ2, and Bmβ3, have no introns.

### Alternative splicing

**Figure 4:**
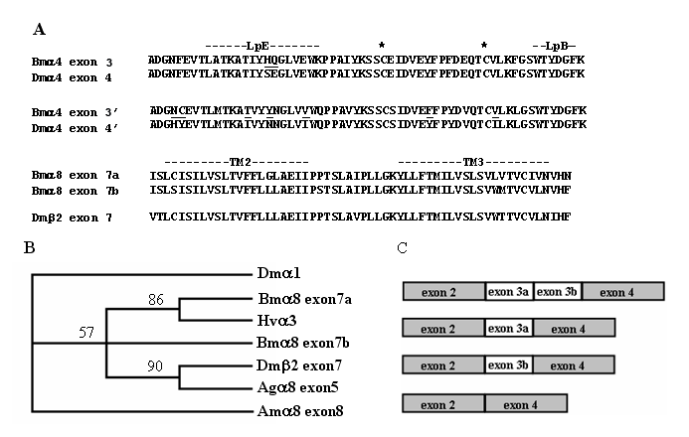
**Alternative splicing of *Bombyx mori *nAChRs**. A) Comparison of corresponding exons of *Bombyx *and *Drosophila *nAChRs. LpE and LpB indicated are loops involved in acetylcholine binding. Two asterisks stand for Cys residues forming disulfide bond separated by 13 amino acid residues. TM2 and TM3 are transmembrane motifs engaged in forming the cation permeable channel. B) Phylogenetic relationship of Bmα8 exon 7s and their corresponding exons from *Heliothis*, *Apis*, *Anopheles *and *Drosophila*. Protein sequence of Dmα1 corresponding to Bmα8 exon 7s is included as an outgroup. Numbers at each node represent bootstrap values with 1,000 replicates. C) Four splicing patterns between exons 2 and 4 of *Bombyx mori nAChR α*6.

*Drosophila *nAChR subunit α4 has two alternative forms differing in residues near the Cys-loop; the α4 subunit with exon 4' displays a less efficient assembling ability than that with exon 4 [22]. Silkworm α4 also has these two alternative exons (Figure 4A). Jin et al (2007) cloned *B. mori *α6 and reported that the exons 3 and 8 of this subunit each possess two alternative forms [18]. *B. mori *α6 could have exon 3a, exon 3b or both in tandem. We found a transcript of Bmα6 missing both exons 3a and 3b (Figure 4C). Alternative splicing of silkworm nAChRs not only occurs in α4 and α6, but also in α8. Silkworm α8 has two alternative exon 7s, located at TM2 and TM3 (Figure 4A). Two alternative exon 4s of α4 are found in *Drosophila*, *Apis*, *Anopheles*, and *Bombyx*. It has been postulated that these alternative exons have arisen from a very ancient duplication event before the evolutionary divergence of those insects. However, we found that the two alternative exon 7s in α8 are extremely similar (86% identity), indicating a more recent duplication event. A phylogenetic tree was constructed using protein sequences corresponding to the exon 7s from several insects, showing that duplication of exon 7s appeared before the divergence of two lepidopteran insects, after the divergence of *Apis *and *Bombyx*, and at the same time with the divergence of *Bombyx *from two dipteran insects (Figure 4B).

### Truncated transcripts

**Figure 5:**
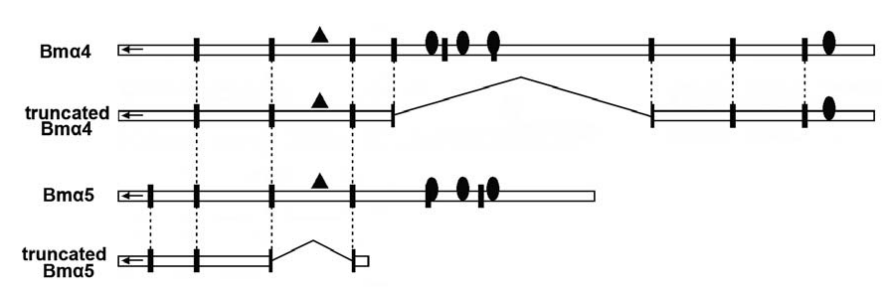
**Comparison of Bmα4, Bmα5, and their truncated transcripts**. Missed exons are displaced by bridge lines. N-terminal signal leader peptides are symbolized by arrows. Exon junctions are indicated by vertical bars. Sites of transmembrane motif are indicated by ovals and Cys-loops are showed by triangles. Identical exon boundaries are showed by dotted lines. A short bar downstream of the bridge line of truncated Bmα5 indicates a premature stop codon because of a frame shift.

Existence of nAChR transcripts lacking some exons or retaining some introns has been reported in many insects as well as in vertebrates [7,9,23]. It was supposed that the translation products of these transcripts may function as acetylcholine "sponge" like molluscan ACh-binding protein [24] or change physiological properties by interfering with the function of normal subunits [23]. In our experiments, truncated transcripts of Bmα4 and Bmα5 were identified (Figure 5). Bmα4^Δ6,7,8 ^lacks exons 6–8 which includes Loop F and TM1–3. Bmα5^Δ4 ^lacks exon 4, which introduces a premature stop codon because of a frame shift and only a very short open reading frame is retained. *D. melanogaster *α4 and α5 also had truncated transcripts, but they lack different exons [7].

### RNA editing

Four *D. melanogaster *nAChR subunits, α5, α6, β1, β2, were found having RNA editing sites. Their *B. mori *counterparts, α5, α6, α8, β1, were examined for RNA editing sites in our experiments. cDNA clones from separate RT-PCRs and the corresponding regions of genomic DNA were sequenced and compared. While the genomic DNA sequences were same as those in the silkworm genome sequence database [19], five bases of α6 (nts #392, 394, 395, 447, and 454) in the cDNA sequence were different from genome DNA sequence, likely resulting from RNA editing. These nucleotide changes result in altered residues of N131S, N132G, I149M, and T152A. Possible RNA editing was also found in three transcript variants of the *B. mori *α6 gene [18]. No RNA editing sites were found in other three subunit genes examined.

## Discussion

With the help of the silkworm genome sequence and by RT-PCR and RACE, we identified the first nAChR subunit gene family in a lepidopteran insect, and the fourth insect nAChR gene family, following *D. melanogaster*, *A. gambiae*, and *A. mellifera*. With 12 members, the nAChR gene family of *B. mori *is the most complex one among those reported for insects. Bmα1–8 and β1 are more similar to the counterparts of other insects compared with the other three subunits, α9, β2 and β3. The phylogenetic relationship of the three subunits indicated that they might have arisen from two separate evolutionary duplication events. The first event likely took place after *Bombyx *had diverged from *Drosophila *and *Anopheles*, and the second might occur after *Bombyx *had diverged from *Apis*.

The GEK motif preceding TM2 is conserved in most insect nAChR subunits, and the glutamate residue is considered to be associated with cation selectivity [2]. Sequence comparison results show that most of the *B. mori *nAChR subunits contained a GEK motif preceding TM2 (Figure 1), while the motif was replaced by STR in α9 and β3, and by TIR in β2. We calculated the amino acid identities of these three subunits with anion channel subunits including *D. melanogaster *GABAR, RDL [GenBank: NM_079267], glutamate-gated chloride channel [GenBank: U58776], histamate-gated chloride channel [GenBank: AF382401], and vertebrate serotonin receptor [GenBank: AJ005205]. *B. mori *α9 shares 10–17% identities with those anion channel subunits, while it shares 27% identity with *Apis *nAChR α9. *Bombyx *β2, β3 share 8–12% identities with above anion channel subunits, they show 17% identities with *A. mellifera *β2. These results show that *Bombyx *α9, β2 and β3 are likely nAChR subunit candidates as opposed to being other members of the cys-loop LGIC superfamily. Whether *Bombyx *α9, β2 and β3 possess the cation selectivity as other GEK-motif-containing AChR subunits remains to be determined.

It is an unusual finding that *B. mori α5 *only has three transmembrane regions, lacking TM4. Our 3' RACE result showed the α5 mRNA contained a consensus polyadenylation signal (AATAAA) located 22 bp upstream of the poly(A) tail. According to the 3'-terminal sequence, we designed gene specific primers and performed additional RT-PCR, but failed to find any longer cDNA product that would include TM4 or another distinct region. These results suggest that *B. mori *α5 contains only three transmembrane regions.

Alternative splicing of several exons results in changes of residues belonging to Loops D, E, Cys-loop, TM2, and TM3. The truncated transcripts lack exons that contain sequences for ligand binding or the ion channel pore. We also detected possible RNA editing events in nAChR genes. It was reported that RNA editing might change during insect development [18], so additional editing sites might exist in α6 or other subunits.

## Conclusion

Our results show the silkworm contains the largest insect nAChR gene family characterized to date, including nine α-type subunits and three β-type subunits. RNA-editing, alternative splicing and truncated transcripts were found in several subunit genes, which might further enhance the diversity of the gene family. These results provide a foundation for future characterization of possibly tissue-specific and developmental expression patterns and the physiological functions of lepidopteran nAChR receptors. Furthermore, the comparison of the nAChR gene family of *Bombyx *with those of other insects, in particular Lepidoptera, may serve as an important basis for developing improved insecticides that spare an economically important insect while controlling major agricultural pests.

## Methods

### Insects

The fertilized *B. mori *(Strain Dazao) parent eggs were kindly presented by Dr. Li Muwang in the Silkworm Research Institute, Agricultural Academy of China, and the larvae were reared with artificial diet at 25°C.

### Bioinformatics analysis and identification of nAChR subunits in the *Bombyx *genome

The *Bombyx mori *genome sequence database [19] was examined with all *D. melanogaster *and *A. mellifera *nAChR sequences using the tBlastn algorithm [25] to produce candidate gene fragments. For each subunit, the gene sequences having the highest similarity were used to design gene specific primers for RT-PCR [see Additional file 1] and RACE [see Additional file 2]. 5' of Bmα7 was obtained by searching the EST database using Blastn method [20].

### RT-PCR

Total RNA was extracted from heads of 4th instar larvae using TRIzol reagent (Dingguo, Shanghai, China) according to the protocol. One μg total RNA was used for synthesizing the first-strand cDNA using PrimeScript™ Reverse Transcriptase (Takara, Dalian, China) and gene specific primers. Subunit-specific oliogonucleotide primer pairs annealing to different exons were designed to generate genomic and cDNA PCR products that differed in size (except for three subunit genes without any intron). Following PCR reactions were performed in a total volume of 25 μL composed of 1 × PCR buffer, 0.2 mM dNTP mix, 1.5 mM MgCl_2_, 1 U *Taq *polymerase (Takara, Dalian, China), 0.4 μM each primer, and 1 μL cDNA template.

Some N-terminal signal peptides and C-termini are highly variable between subunits, and could not be identified with confidence by BLAST. In these cases, 5'- and 3'-RACE were performed using the SMART™ RACE cDNA Amplification Kit (Clontech, USA) to obtain the complete ORF sequences. For 5'-RACE, the cDNA was synthesized using gene specific primers. For both 3'-RACE and 5'-RACE, following PCR amplification we used another round of nested PCR. Other PCR conditions refer to the manual.

All PCR products were analyzed by electrophoresis in 1% agarose gel, and DNA fragments were purified with the DNA Fragment quick purification/recovery kit (Dinguo, Shanghai, China). The purified DNA fragments were ligated into pMD 18-T (Takara, Dalian, China), and sequenced with an ABI-3730 automatic sequencer. The sequences reported in this paper have been deposited in GenBank with accession numbers: Bmβ1 [GenBank: EU082071]; Bmβ2 [GenBank: EU082072]; Bmβ3 [GenBank: EU082073]; Bmα1 [GenBank: EU082074]; Bmα2 [GenBank: EU082075]; Bmα3 [GenBank: EU082076]; Bmα4 variant 1 [GenBank: EU082077]; Bmα4 variant 2 [GenBank: EU082078]; Bmα4 variant 3 [GenBank: EU082079]; Bmα5 variant 1 [GenBank: EU082080]; Bmα5 variant 2 [GenBank: EU082081]; Bmα6 variant 2 [GenBank: EU082082]; Bmα6 variant 4 [GenBank: EU082083]; Bmα7 [GenBank: EU082084]; Bmα8 variant 1 [GenBank: EU082085]; Bmα8 variant 2 [GenBank: EU082086]; Bmα9 [GenBank: EU082087].

### Sequence and phylogenetic analyses

Signal peptide cleavage sites were predicted using the SignalP 3.0 server program [26]. Transmembrane regions were speculated using the TMpred program [27]. Potential post-translational modification sites including those for N-glycosylation, cyclic AMP (cAMP)-binding, and protein kinase C (PKC), CK2, and tyrosine kinase phosphorylation were identified using the PROSITE database [28]. All *B. mori *nAChR subunit protein sequences were aligned using software ClustalX [29] and edited with software GeneDoc [30]. Similarity and identity values between nAChR sequences were the output from the GeneDoc program. All *Drosophila*, *Apis*, *Anopheles*, and partial lepidopteran nAChR subunit protein sequences were aligned with ClustalX and the multiple alignment result was used as input into the PAUP software (v.4.0b10) to construct phylogenetic trees with the maximum parsimony method and bootstrapping sampled for 1,000 times [31], and the tree was viewed with TreeView application [32].

## Authors' contributions

YMS carried out the molecular cloning and analysis of the sequences and drafted the manuscript. KD helped to design the study and draft the manuscript. CXZ conceived of the study, and participated in its design and coordination and helped to draft the manuscript. All authors read and approved the final manuscript.

## Supplementary Material

Additional file 1 Sequences of RT-PCR primers used in this study. All sequences are showed in 5'→3' direction. Click here for file

Additional file 2 Sequences of RACE primers used in this study. One gene specific primer and one universal primer are used in each round of PCR. Only gene specific primers are listed and universal primers are UPM in first round PCR and nested NUP in second round PCR according to the user manual. All sequences are showed in 5'→3' direction. Click here for file
