# Supplementary material for: The nicotinic acetylcholine receptor gene family of the silkworm, Bombyx mori
Source: BMC Genomics. 2007 Sep 15;8:324. doi: 10.1186/1471-2164-8-324 (PMC2045683; doi:10.1186/1471-2164-8-324)
Supplement: Additional file 1 — Sequences of RT-PCR primers used in this study. All sequences are showed in 5'→3' direction. [file 1471-2164-8-324-S1.pdf]

Additional file 1

Sequences of RT-PCR primers used in this study*

Subunits	Reverse transcription primers	PCR primer pairs Forward	PCR primer pairs Reverse	Product length
α1	cacagtgagagataggagg	gtggtgttagcctaagatgg	gccgtcgtggtggaggatcgcctt	415
	cacagtgagagataggagg	agtgacgataatgacgaagg	gattgactgtgtagaacagag	374
	gagctcccagaacgtctt	ctcggaccacattggaatgg	ggccgaagacgtaagacgaa	1070
α2	tgctttccaatgtaccgagg	tgagagacacgagaggta	gtcttatgctataagactcggc	1202
	tacggttcctgacagca	tcccttggagtatggtgg	actcgcgaaggtcgatacc	1324
	tacggttcctgacagca	tctcgggcgtgctgtcaa	actcgcgaaggtcgatacc	747
α3	gagcatgttgctcgtaatagtgg	atgggtggaacagacttgg	gccgcatcgtgatattga	488
	tcaaaaaaaaattcgacttag	gctccacaaggcaatcga	aattcgacttagattcgatc	390
	gccgcatcgtgatattga	aacgcgccatgcgctgac	tcgcaggaggacttgtagat	490
	aattcgacttagattcgatc	ggacatactcgaggttcctg	gaagatccacaggaacagt	769
α4	gtgacctgaagtggacgtt	tgaggtcgcgagctgatg	gtggacgttgagaacgacca	996
	ttgttattaatgtacgtatctttg	ccgagttctacatgtcagt	ggaaggttgagatttacatatcg	1148,695
	gtgacctgaagtggacgtt	gaagtgacgctgatgacgaa	gtggacgttgagaacgacca	621
	gtgacctgaagtggacgtt	cgaccctttacgtaacgatg	gcacacaggtttgcacgt	527
α5	tgaccgtcttcctgatgac	gcgatggaacactagtgacttcga	cacgtagaacatcggtctcctacg	267,451
	ggaaattctttgcctcgag	gtcagcttcgaggctatcagacat	cgagaggcgactgcttaatgtgtt	550
α6	gtgagcatgcacatgagca	agacgaacgtggtggtcaga	gtaccgtctcactgcacgat	1144
	ccaccggcttcgtccttc	gtgtcggcagcgtatgag	gggcacgtacagacaact	446,491
α7	gtattcgtggtatcacag	ggtttccattcgatgacca	cgtttcgcatgcgtatccgca	1035
	cacggaagaggcgaccat	atctgctggaccactacaa	ccaactgccaaacttcat	419
α8	tctggaagcgagtac	cttggtctgcgtagcgtatcg	ggttagtagtcatgacctga	344
	tctggaagcgagtac	ctcgaagcgaatcctgatg	gtcctcctcatcatcaagag	1019
	tctggaagcgagtac	ctcgaagcgaatcctgatg	atgcacgtttactatgcac	930
	ggttagtagtcatgacctga	cgctcagcgtattggtca	ctgaaatacatatcttccgttcg	673
	ctgaaatacatatcttccgttcg	cgctcagcgtattggtca	gcaccattgacatgaacttcc	462
α9	gctgatctaagcgaatgg	cgcaaacatgaaactgtacgtag	gacgcagaggttggttca	1448
β1	ccagaatactgacagt	ggtgttcagaagacgaagaac	cgataccagaagcaggaacac	785
	ttaatcagttactatctgac	catgggatatcatagaagtac	caatgtagaatggcatactac	978
β2	gcatggatgtctctgcac	ctcagtacgaccacagct	cacacgaaatgccttgcat	1200
β3	tgagcactcggaaggtct	cgaggagaccgtacttgtca	acgagatagaactcgtcg	941
	tgagcactcggaaggtct	accggatcgtatgttggat	tgagcactcggaaggtct	405

*All sequences are showed in 5’→3’ direction
