# Supplementary material for: The nicotinic acetylcholine receptor gene family of the silkworm, Bombyx mori
Source: BMC Genomics. 2007 Sep 15;8:324. doi: 10.1186/1471-2164-8-324 (PMC2045683; doi:10.1186/1471-2164-8-324)
Supplement: Additional file 2 — Sequences of RACE primers used in this study. One gene specific primer and one universal primer are used in each round of PCR. Only gene specific primers are listed and universal primers are UPM in first round PCR and nested NUP in second round PCR according to the user manual. All sequences are showed in 5'→3' direction. [file 1471-2164-8-324-S2.pdf]

Additional file 2

Title: Sequences of RACE primers used in this study*

Subunits	Gene specific primers	
	First round	Second round
β1 5’RACE	cgccagtgatacttggtccccattgaac	caaacgcaagaccaaatctcacatccact
α5 5’RACE	cacgtagaacatcggtctcctacg	cctgaaatccgtcgtaggtccaaga
α5 3’RACE	gcgatggaacactagtgacttcga	gtcagcttcgaggctatcagacat

* One gene specific primer and one universal primer are used in each round of PCR. Only gene specific primers are listed and universal primers are UPM in first round PCR and nested NUP in second round PCR according to the user manual. All sequences are showed in 5’→3’ direction.
